# Gut microbiota-dependent trimethylamine n-oxide pathway contributes to the bidirectional relationship between intestinal inflammation and periodontitis

**DOI:** 10.3389/fcimb.2022.1125463

**Published:** 2023-01-13

**Authors:** Qiqi Wang, Yue Sun, Tianyu Zhou, Cong Jiang, Lan A, Wenzhou Xu

**Affiliations:** ^1^ Department of Periodontology, School and Hospital of Stomatology, Jilin University, Changchun, China; ^2^ Department of Oral Implantology, School and Hospital of Stomatology, Jilin University, Changchun, China; ^3^ Jilin Provincial Key Laboratory of Sciences and Technology for Stomatology Nanoengineering, Changchun, China

**Keywords:** trimethylamine N-oxide (TMAO), periodontitis, gut microbiota, intestinal-oral axis, inflammation

## Abstract

**Background:**

Intestinal inflammation and periodontitis influence the development of each other through the bidirectional relationship. As the intestinal microbiome metabolite, trimethylamine-N-oxide (TMAO) could contribute to chronic inflammation in the gut by influencing the gut microbial composition and intestinal immunity. Increased circulating TMAO levels often accompany clinical findings in patients with experimental periodontitis. However, the role of TMAO in the bidirectional relationship between intestinal inflammation and periodontitis remains unclear. Thus, we explored whether TMAO influences the periodontitis process by affecting intestinal immunity and microbial composition in this article.

**Methods:**

Periodontitis was induced by unilateral ligation of the first molar in mice, and 3,3-dimethyl-1-butanol (DMB) was used as an inhibitor to reduce TMAO circulating. Twenty-five BALB/c mice were randomly assigned to five study sets (n = 5/group): no periodontitis with DMB (Control group), periodontitis (P) group, periodontitis with TMAO (P+TMAO) group, periodontitis with TMAO and DMB (P+TMAO+DMB) group, and periodontitis with DMB (P+DMB) group. The effect of TMAO was determined by assessing changes in intestinal histology, intestinal flora composition, periodontal tissue, and periodontal pro-inflammatory factors at ten days.

**Results:**

The outcomes indicated a marked improvement in the intestinal inflammation severity, and intestinal flora diversity was reduced. *Firmicutes* number and the ratio of *Firmicutes/Bacteroidetes* were improved in the P+TMAO group. In addition, the alveolar bone resorption and the degree of periodontal tissue inflammation were more severe in the P+TMAO group than in other groups. Immunohistochemistry showed higher levels of TGF-β and IL-1β expression in the periodontal tissues of P+TMAO.

**Conclusions:**

Our data suggest that TMAO could influence periodontal immunity and promote periodontal inflammation by affecting the intestinal microenvironment, revealing TMAO may affect the development of periodontitis through the bidirectional relationship of the oral-gut axis.

## 1 Introduction

Periodontitis, which affects up to 10% of people in severe cases, is an immune-related disease caused by the interaction between oral bacteria and immune cells in biofilm, resulting in periodontal tissue damage (Nicu et al., 2009; Gonzales, 2015; Herrera et al., 2022). Since periodontitis is the result of local immune imbalance, many systemic disorders, such as rheumatoid arthritis (RA) and systemic lupus erythematosus (SLE), have been found to be associated with periodontitis (Sete et al., 2016). In recent years, gastrointestinal disorders related to immunity, like inflammatory bowel disease (IBD), have also been reported to be related to periodontitis (Kitamoto et al., 2020b; Sete et al., 2016; Zhou et al., 2021).

Oral and gastrointestinal mucosa is continuous with each other, and both are associated with microbiology and immunology (Kitamoto et al., 2020b). *Firmicutes* and *Bacteroidetes*, which make up over 90% of the entire population, are the two main dominating microbiota in the human digestive tract, and subdominant microbiota includes *Proteobacteria*, *Actinobacteria*, and *Verrucomicrobia*, which regulate physiology, metabolism, immunity, and health-disease processes (Vieira et al., 2016; Fan and Pedersen, 2021). Interestingly, the human oral is made up of six major germ classes, including *Actinobacteria*, *Bacteroides*, *Firmicutes*, *Fusobacteri*a, *Proteobacteria*, along with *Spirochetes* (Xu et al., 2015). When periodontitis occurs in the oral cavity, it results in the expansion of the *Klebsiella*/*Enterobacter* species, which can be transferred to the lower digestive tract and colonized (Kitamoto et al., 2020b; Magne et al., 2020).

The result of chronic intestinal inflammation depends on the makeup of the gut microbiota, immunological response, host genetic factors, and how these variables interact. Cross-talk between the gut microbiota and the host immune system can either prevent or mitigate this condition (Cao, 2017). Studies have shown that colonization of the gut by oral bacteria can be engaged in the progression of intestinal inflammatory diseases by interfering with intestinal flora composition and intestinal immunity (Cao, 2017; Kitamoto et al., 2020b). Oral microorganisms can mediate intestinal inflammation by invading the intestinal epithelium, causing the release of inflammatory factors, decreasing the killing capacity of macrophages and natural killer (NK) cells, and promoting pro-inflammatory Th 1 and Th 17 responses (Kitamoto et al., 2020b; Zhou et al., 2019; Read et al., 2021), and there is growing evidence showed that in the pathophysiology of gastrointestinal illnesses, the “oral-intestinal axis” might be crucial (Kitamoto et al., 2020b). However, fewer studies have been conducted to survey the impact of microecological changes in gut flora on periodontitis progression, establishing a brand-new avenue for periodontitis clinical therapy.

Currently, it has been hypothesized that the progression of colitis and periodontitis may be influenced by the invasion of CD4^+^T cells (Zhang et al., 2021). It is widely accepted that immune cell infiltration of mucosal tissues is a hallmark of periodontitis. T cells are its main immune cell population, which is related to the expression of inflammatory cytokines (Gonzales, 2015; Williams et al., 2021). In periodontitis, dendritic cells(DCs) capture and process antigens and express the costimulatory molecules and cytokines needed for antigen presentation to B and T cells. DCs also play an important role in the “tolerance” of T cells to autoantigens, thereby reducing the risk of autoimmune reactions. Meanwhile, DCs are an effective stimulant for NK cells. NK cells, a unique subgroup of cytotoxic T lymphocytes, are abundant in periodontitis lesions, and NK cell activation has a causal relationship with periodontal tissue destruction (Krämer et al., 2013; Meyle and Chapple, 2015). Moreover, bacterial invasion of periodontal tissue leads to CD4^+^ T cells that differentiate into Th17 cells, which protect our body against pathogens by producing mucosal immune response and bone damage (Tsukasaki et al., 2018). Thus, T cells may be the bridge between intestinal inflammation and periodontitis. However, the immune response of T cells may be influenced by the nutritional microenvironment, such as oxygen, glucose, and microbial metabolites (Ramsay and Cantrell, 2015). The immune system’s metabolism can be influenced by metabolites produced by the intestinal microbiota, with TMAO, short-chain fatty acids (SCFAs), and bile acids (BAs) being particularly significant. (Michaudel and Sokol, 2020). TMAO, a toxin generated from gut bacteria and derived from nutrients such as dietary choline, has been found to be associated with immunological disorders. For instance, TMAO raises the risk of death from chronic kidney disease, nonalcoholic fatty liver disease, and colorectal cancer (Wang et al., 2020a; Fan and Pedersen, 2021; Zhang et al., 2022). Studies have shown that long-term TMAO supplementation can cause significant changes in the microbial composition of the entire cecum leading to the activation of the intestinal immune system and chronic inflammatory illnesses progression (Zhu et al., 2016; Atarashi et al., 2017). Simultaneously, clinical findings in patients with periodontitis are often accompanied by increased circulating levels of TMAO (Xiao et al., 2021; Zhou et al., 2022). Accordingly, TMAO may play an essential role in periodontitis and intestinal inflammation progression. On the one hand, by activating NF-κB, boosting the expression of inflammatory genes (IL-1), as well as enhancing oxidative stress, TMAO could enhance the indication of inflammatory markers (Yang et al., 2019). On the other hand, TMAO has also been correlated with the improved efficacy of immunotherapy in the clinical cohort (Wang et al., 2022b). TMAO enhances M1 macrophage polarization through NLRP3 inflammasome activation, and polarized macrophages participate in the effect on periodontal osteoclast by TGF-β (Jiang et al., 2019; Wu et al., 2020; Wu et al., 2021).

Up to now, no researchers have focused on the role of TMAO in the relationship between intestinal inflammation and oral inflammation. The widespread presence of TMAO in daily red meat is a growing concern not only for individual health but also for the health of the planet. Therefore, we designed a series of follow-up experiments to prove that TMAO can affect the process of periodontitis. In this study, we used DMB, a structural analog of choline, which nonlethally reduces the TMAO concentration in mice (Wang et al., 2015). By administering exogenous TMAO and its inhibitor DMB to mice, we observed the changes in periodontal tissue, intestinal tissue, and intestinal flora in mice with different periodontitis. Further proved that TMAO affected the development of periodontitis by involving the regulation of the intestinal-oral axis.

## 2 Material and methods

### 2.1 Animals and dietary treatment

All of the male, 6–8 week old, specific pathogen-free (SPF) grade BALB/c mice used in this investigation came from Liaoning Changsheng Biotechnology Co., Ltd. (Benxi, China). Twenty-five BALB/c mice were maintained in a strict 12 h/12 h light/dark cycle at room temperature (18–24°C) and comfortable humidity (50–60%). Mice were given a starting body weight of 18:2 ± 1:1 g, supplemented with adequate water and the same food. Before the induction of periodontitis, all mice underwent one week of acclimatization. The Institutional Animal Care and Use Committee (IACUC) of Jilin University (Changchun, China) authorized animal experiments (BALB/c). The IACUC Ethics Committee considered the full proposal and approved the animal care and use permit license. Classify twenty-five BALB/c mice randomly into five sets (n = 5/group): No periodontitis with DMB (Control) group, periodontitis (P) group, periodontitis with TMAO (P+TMAO) group, periodontitis with TMAO and DMB (P+TMAO+DMB) group, periodontitis with DMB (P+DMB) group. DMB and TMAO were bought from Shanghai Aladdin Biochemical Technology Co., Ltd. in China. Periodontitis was induced *via* unilateral ligation in the first molar position in mice. All mice were fed standard chow and randomized to receive free access to plain water, water complemented with 1% water (v/v, TMAO), or water completed with 1% (v/v, TMAO+DMB) or 1% (v/v, DMB). Fresh bottles of water were available every day. All sets’ drinking water and food consumption were studied and controlled daily. Within 10-day feeding, kill all mice by pentobarbital sodium overdose. After removing the mandible, the maxilla was separated along the mid-palatal suture, then immobilized the right maxilla in 4% paraformaldehyde for 48 h. In addition, the distal ileum was cut into 1 cm segments and anchored in 4% paraformaldehyde last 48 hours.

### 2.2 Histological staining and histopathological evaluation of ileum

To observe the changes in intestinal tissue, make the immobilized tissue into 5-mm paraffin cuts after dehydration and encapsulation. After hematoxylin-eosin (HE) staining, morphological variations were noted under the optical microscope. To assess the histological changes in the ileal tissue, HE-stained inflammatory cells were counted in the fixed area (1× 10^4^ μm^-2^) at a magnification of × 400, which were recognized as immune cells based on shape, size, and position. The visual field’s squamous epithelial cells and fibroblasts were not counted. The overall percentage of inflammatory cells indicated the presence of inflammation in the field of view (Zhao et al., 2022).

### 2.3 Bioinformatics analyses

Microbial DNA was extracted from the distal ileum through the Power Soil^®^ DNA Isolation Kit (MoBio Laboratories, CA, USA) based on the instructions of the manufacturer to determine any changes. After sequencing, QIIME2 was used to quality-filter raw reads to remove low-quality reads (2019.4). With a cutoff similarity of 97%, UPARSE grouped the acquired high-quality, unique orders into operational categorization units (OTUs). The Ribosomal Database Project (RDP) classifier used a reliability threshold of 70% to examine each 16S rRNA gene sequence taxonomy against the silva (SSU132) ribosomal RNA gene database. Refraction and alpha diversity (abundance: Chao 1 and observed species indexes; diversity: Simpson and Shannon and Faith indexes; evenness: Pielou Index) were calculated using Mothur (V 1.30.2). Principal component analysis (PCA) was on the grounds of species abundance matrix using R language. The Venn plot was generated from the ASV/OTU abundance table. The key genus differing significantly among different groups was shown in STAMP. The specific bacteria were identified using linear discriminant analysis (LDA) scores (LDA score >2.0 and P < 0.05), which were derived from the linear discriminant analysis effect size. Spearman’s correlation was used to determine relationships between the relative abundance of the main genera and the associated metabolic indices.

### 2.4 Micro-computed tomography imaging and bone resorption analysis

The maxillary biopsy tissue of each group fixed in 4% paraformaldehyde was examined by a microcomputer tomography (μCT50) system (SCANCO, Switzerland). On the basis of the manufacturer’s procedures, three-dimensional (3D) digitized images of the palatal view were collected for each sample using 3D reconstruction software (Analyze12.0, AnalyzeDirect, Overland Park, KS, 66085, USA). Alveolar bone loss was analyzed from 3D mode. From the crest of the alveolar bone to the cemento-enamel junction (CEJ), linear measurements were recorded (ABC) according to the tomographic method (He et al., 2020).

### 2.5 Histopathological evaluation of jaw bone histology

After scanning, the specimens were decalcified, dewatered, and embedded in paraffin. Then, they were mounted in the medial plane on 4-μm serial sections on anti-adhesive slides in preparation for HE staining, Masson staining, as well as immunohistochemical (IHC) staining. IL-1β primary antibody (dilution 1:1000, Abcam, USA) and TGF-β primary antibody (dilution 1:1000, Abcam, USA) was used for IHC staining. Three consecutive square fields involving junctional epithelium (JE), the gingival epithelium (GE), and alveolar bone (AB) in the mesial coronal area of the first molar were selected from each section. Six sections of each group were analyzed, and each measurement was counted by the examiner in a blind manner.

### 2.6 Statistical analysis

The SPSS 18.0 program was used to perform all statistical analysis. Express the data by the mean and standard deviation (SD). In addition, they were analyzed by one-way ANOVA and Student t-test. PCA analysis based on the species abundance matrix with downscaling was done to assess the changes in gut microbial community structure in different dietary treatments. To identify bacterial taxa that differed in abundance between sets, we conducted the linear discriminant analysis (LDA) effect size (LEfSe)analysis. Make p<0. 05 the boundary of statistical significance (Cisbani and Bazinet, 2020; Wang et al., 2020a).

## 3 Results

### 3.1 TMAO promotes intestinal inflammation in mice with periodontitis

**Figure 1:**
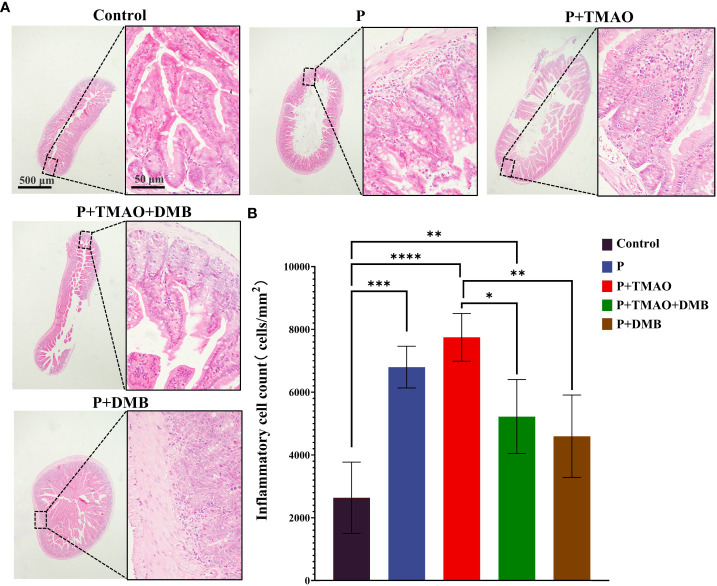
TMAO promotes intestinal inflammation in mice with periodontitis. **(A)** Representative pictures of intestine tissue by HE dyeing. **(B)** Quantification of monocytes, macrophages, lymphocytes, and neutrophils in intestinal HE samples.. The symbols *, * *, * * *, and * * * * have the meanings P ≤ 0.05, P ≤ 0.01, P ≤ 0.001, and P ≤ 0.0001, respectively.

Previous studies have found that the changes in the plasma level of TMAO were related to many undesirable host diseases, including nonalcoholic steatohepatitis (NASH), CVD, IBD, and central nervous system diseases (Hosseinkhani et al., 2021). To investigate the association between TMAO and intestinal inflammation, we analyzed the histomorphology and the quantity of ileal inflammatory cells in each set. The tight junction of the ileum was widened in the group of P+TMAO *via* the HE staining results, accompanied by an increase of inflammatory cells (**Figure 1**). However, the use of DMB significantly reversed the adverse effects of TMAO on tissue damage and inflammation (**Figure 1**). Moreover, compared with Control and P+DMB groups, the intestinal tissue of the P group showed mild damage to the intestinal epithelium, with a small amount of intestinal villous defects, a vascular proliferation of submucosal cells, increased erythrocytes, moderate inflammatory cell infiltration in the lamina propria, and reduced glands (**Figure 1A**). Interestingly, the intestinal HE in the P+TMAO group showed sparse and broken intestinal villi, atrophy, irregular shape, severely damaged intestinal gland damage, deepened crypt, submucosal congestion, and edema with extensive inflammatory cell infiltration (**Figure 1A**). Compared with the P+TMAO group, the degree of intestinal inflammatory damage was considerably lower in the P+TMAO+DMB group. Consistently, we found significantly higher inflammatory cells in the P+TMAO group than in the other four groups, which was statistically different from the Control, P +TMAO+DMB group, and P +DMB group (**Figure 1B**). Additionally, there was a statistical difference in the number of inflammatory cells in the Control group compared with P and P+TMAO+ DMB groups. These results suggest that TMAO may contribute to intestinal inflammation.

### 3.2 TMAO contributes to the changes in the components and abundance of intestinal flora in mice with periodontitis

**Figure 2:**
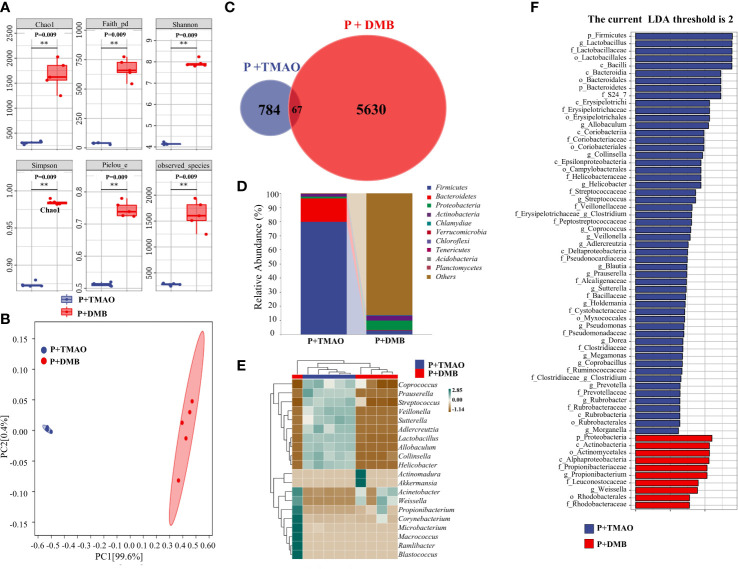
Difference of intestinal flora composition between the P+TMAO group and P+DMB groups. **(A)** The value of the indicated index of alpha diversity in the P+TMAO and P+DMB groups. The symbol ** has the meaning P ≤ 0.01. **(B)** PCA plot of microbial composition. **(C)** Venn graph based on ASV/OTU. In the figure, each color block represents a group, the overlapping area between the color blocks indicates the ASV/OTU shared by the corresponding group, and the number of each block indicates the number of ASV/OTU contained in the block. **(D)** The stack bar plot showed the composition of microbiota at the phylum level. **(E)** The heat map of the top 20 genera showed trends in species abundance distribution trends across samples. **(F)** LDA scores of intestine tissue samples.

The typical mucosal immune system monitors the intestine’s microbial makeup. Intestinal disorders are caused by inflammation brought on by aberrant immune responses, affecting intestinal microbiota balance (Shi et al., 2017). Therefore, we compared the intestinal microflora between the P+TMAO group and the P+DMB group. Through genome sequencing, we examined the samples’ components and amounts of gut bacterial flora. We observed that diversity (Simpson and Shannon and Faith indexes), richness (Chao1 and observed species indexes), and evenness (Pielou index) were lower in the P+TMAO group compared with the P+DMB group after 10-day treatment (**Figure 2A**). Furthermore, the tissue samples of the two groups showed significant separation in PCA (**Figure 2B**). According to the Venn diagram, the P+TMAO group contains 851 ASV/OTU, and the P+ DMB group contains 5697 ASV/OTU, while only 67 ASV/OTU are shared between the two groups (**Figure 2C**). These results imply that TMAO has led to a decrease in intestinal flora species. In addition, to compare the flora composition variations in flora composition among the two sets, we analyzed the trends in the distribution of the phylum, genus, and species. *Firmicutes* were substantially abundant in the P+TMAO group versus the P+DMB group at the phylum level (**Figure 2D**). Interestingly, the phylum content of *Firmicutes* and *Bacteroidetes* was 79.58% and 16.38% in the P+TMAO group. The proportion of *Firmicutes/Bacteroidetes* in the P+TMAO group was obviously improved. Nevertheless, compared to the P+TMAO group, *Firmicutes* abundance of the P+DMB group decreased, while the abundance of *Proteobacteria* was higher. At the genus level, *Coprococcus*, *Prauserella*, *Streptococcus*, *Veillonella*, *Sutterella*, *Adlercreutzia*, *Lactobacillus*, *Allobaculum*, *Collinsella*, and *Helicobacter* increased significantly in the P+TMAO group (**Figure 2E**). Finally, LEfSe analyses were performed to identify intestinal microflora between the P+TMAO group and the P+DMB group. As seen in **Figure 2F**, *p_Firmicutes* and *g_Lactobacilllus* were abundant in the P+TMAO group, whereas *p_proteobacteria* and *c_Actinobacteria* were abundant in the P+DMB group. These results indicated that TMAO might affect intestinal microflora balance *via* inflammation caused by the abnormal immune response.

### 3.3 TMAO affects periodontal tissue inflammation through periodontal immunity

**Figure 3:**
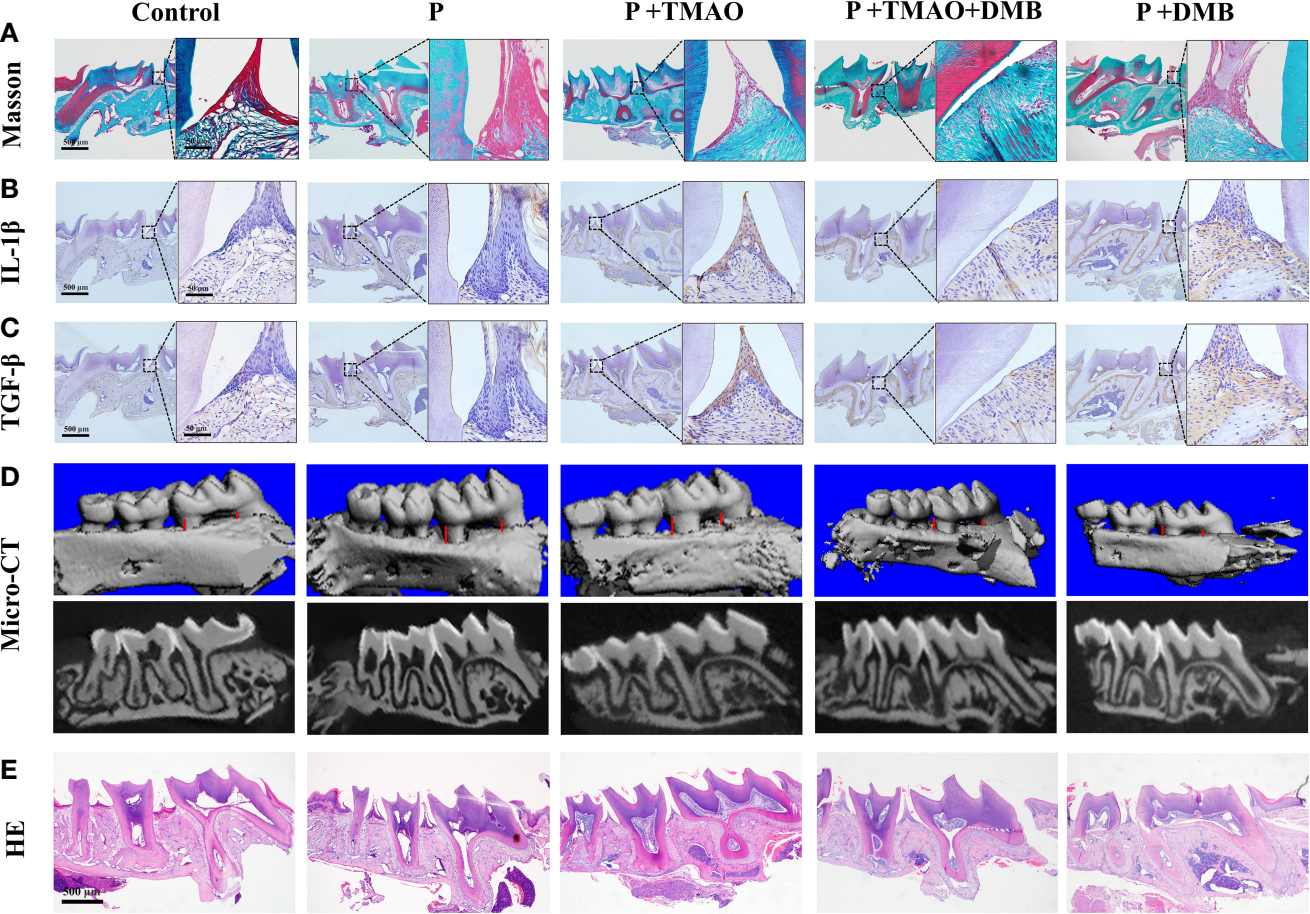
Changes of periodontal tissues and IL-1β as well as TGF-β expression in every group. **(A)**Pictures of alveolar bone at the first molars by Masson dyeing. Red is the gingival epithelium, and blue is the periodontal collagen fibers. **(B)** Photographs of immunohistochemical staining of alveolar bone for IL-1β. **(C)** Images of immunohistochemistry staining of alveolar bone for TGF-β. **(D)** Alveolar resorption at the interproximal sites of maxillary first molars (buccal view) in a reconstruction from Micro–computed tomography (Micro CT) (red line). **(E)** HE staining of alveolar resorption at root furcation (black line).

The periodontal collagen fibers stained by Masson were observed to assess the extent of periodontal tissue damage. There was significant degradation of collagen fibers and infiltration of inflammatory cells among fibers in the P+TMAO group (**Figure 3A**). In addition, weak expression of inflammatory factors IL-1β and TGF-β in the basal layer of epithelial cells was shown in both the Control and P+DMB groups. However, the expression of IL-1β and TGF-β was widespread in the P+TMAO group, and the highest level was in the basal and spiny layers (**Figures 3B, C**). Furthermore, the gingival epithelium, bound epithelium, and alveolar bone were normal in the Control and P+DMB groups. But there was obvious root-migration of bonded epithelium, attachment loss, and alveolar ridge absorption in the P group, P+TMAO group, and P+TMAO+DMB group. In summary, DMB inhibited the effect of TMAO on periodontal tissue inflammation (**Figures 3B, C**). These results suggest that TMAO affects periodontal immunity by inducing the expression of IL-1β and TGF-β, leading to periodontal tissue damage.

### 3.4 TMAO promotes alveolar bone resorption in mice with periodontitis

TMAO could induce intestinal immune response and inflammation through intestinal ecological dysregulation in mice, making it possible to affect the periodontitis process. Likewise, there was evidence that experimental periodontitis is often accompanied by increased TMAO levels in the blood (Xiao et al., 2021; Zhou et al., 2022). To further confirm this hypothesis, the results of micro-CT were analyzed separately (Xiao et al., 2021). We weigh the distance between the buccal roots of the first molars’ typical enamel bone boundary and the top alveolar ridge to estimate the alveolar bone resorption. The P+TMAO group showed significant alveolar resorption and apparent root bifurcation, which were more severe than other groups. Compared to the P+TMAO group, mice in the P+TMAO+DMB group had slower alveolar bone resorption due to ingesting DMB. Moreover, there was no remarkable variation among the Control group and P+DMB group, with periodontal tissues in a healthy state (**Figure 3D**). These results suggest that the inhibition of TMAO may slow down the alveolar bone resorption in periodontitis to some extent. Meanwhile, HE-stained sections showed that the loss of attachment of connective tissue, disruption of collagen fibers, and inflammatory cells infiltration was more severe in the P and P+TMAO groups (**Figure 3E**), implying that exogenous TMAO promoted alveolar bone resorption in mice with periodontitis.

## 4 Discussion

TMAO, a pro-inflammatory metabolite, has been found to be involved in inflammation, inflammation-related immunity, and other mechanisms in diseases such as IBD (Hosseinkhani et al., 2021; Zhou et al., 2022). The intestinal tract promotes the production of ILC3 by IL-2 regulatory Treg cells through the microflora and IL-1β dependent axis, thereby maintaining intestinal immunity (Zhou et al., 2019). But the colonization resistance of intestinal microbiota can be destroyed by TMAO, allowing oral microorganisms to invade and disrupt the intestinal barrier, thus promoting intestinal inflammation (Atarashi et al., 2017; Kitamoto et al., 2020b; Zhu et al., 2016). Consistent with these reports, by analyzing the intestinal barrier through morphological assessment of intestinal villi, we discovered that the intestinal barrier of the P+TMAO group was severely compromised, as evidenced by the group’s shorter intestinal villi and deeper intestinal crypts.

*Firmicutes* and *Bacteroidetes* comprise more than 90% of the entire bacterial community, plus the subdominant phyla *Proteobacteria*, *Actinobacteria*, and *Verrucomicrobia* constitute the vast majority of the human intestinal microbiota (Magne et al., 2020). Recently, dietary choline has been linked to changes in intestinal bacteria and even intestinal ecological problems, including major alterations in the abundance, diversity, metabolism, and function of microorganisms (Koay et al., 2021). Reduced variety is typically a sign of an unbalanced gut microbiota, with an increase in pathogenic bacteria and a decrease in helpful bacteria (Li et al., 2021a). As the choline metabolites, TMAO usually comes from L-carnitine, phosphatidylcholine, and choline, which are found in red meat, fish, eggs, as well as high-fat dairy products. Global analyses of community structure revealed distinctions between chow-fed (0.07% choline) and those fed high choline (1.0%) diets. These differences were more obvious in the presence of DMB. Studies have shown that aging will increase TMAO levels in peripheral and central tissues, induce intestinal microbiota imbalance and decrease α-diversity, while DMB can effectively reduce TMAO levels and improve peripheral metabolism (Wang et al., 2015; Lanz et al., 2022). This is consistent with our experimental results that TMAO can change the structure of intestinal flora and even cause intestinal flora imbalance. Intestinal flora imbalance is also associated with a variety of pathological conditions, for example, the digestive tract, including irritable bowel syndrome and diarrhea; the immune system, including multiple sclerosis, inflammatory bowel disease, and rheumatoid arthritis; the central nervous system, including Parkinson’s disease and Alzheimer’s disease and autism, as well as host energy metabolism including type 2 diabetes, obesity, and atherosclerosis (Magne et al., 2020).

Although it remains unclear whether gut flora dysbiosis is a begetter or a result of the above diseases, the ratio of phylum *Firmicutes*/*Bacteroidetes* (F/B), which expresses how the two dominant phyla are related to one another, has been demonstrated to be linked to several pathological conditions (Magne et al., 2020). The average ratio of F/B of humans is 2.6. An increased F/B ratio has been identified as the marker of a pro-inflammatory state and immune imbalance in autoimmune diseases (Chen et al., 2021a; Yang et al., 2015; Magne et al., 2020), such as obesity, diabetes, and cardiovascular disease. Moreover, the study found a difference in the major group F/B ratio and the gut microbial composition between obese and lean individuals (Wang et al., 2020b). The entire constituent of the gut microbiota is altered in obese patients, and studies have found an increased F/B ratio in hereditary obese mice and mice with high-fat diets (de Sant'Ana et al., 2019). Jun L et al. proposed that TMAO concentrations were associated with the overall microbial compositions. The abundance of 10 bacterial species was significantly correlated with plasma TMAO concentration, including 8 *Firmicutes* species, one *Bacteroidetes* species, and one *Actinobacteria* species (Li et al., 2022). Consistent with previous findings, we found an increase in the abundance of the *Firmicutes* phylum in the P+TMAO group, indicating that TMAO could influence the intestinal microenvironment by affecting the formation and abundance of intestinal microorganisms.

Similar to the findings of Wang X. et al., the decreased abundance of *Firmicutes* was positively connected with the TMAO level in our study (Wang et al., 2020a). Interestingly, we discovered a noticeable reduction in the richness of *Firmicutes* and an abundance of *Proteobacteria* increased in the P+DMB group. DMB affects the liver and intestinal metabolism by decreasing choline levels, which in turn affects the structure and function of intestinal flora, such as decreasing the abundance of *Firmicutes* (Zeisel and da Costa, 2009; Trebicka et al., 2021). Choline is critical in synthesizing acetylcholine, methylation, and gene expression of liver and muscle function (Arias et al., 2020). Even if endogenous choline synthesis is possible, it is insufficient to suit human demands. Many diseases related to intestinal microorganisms, such as atherosclerosis, nonalcoholic fatty liver disease, lipoprotein secretion, and even nervous system problems, are considered to be affected by choline deficiency (Zeisel and da Costa, 2009). Furthermore, *Proteobacteria* are closely associated with hepatic and intestinal metabolism. For example, the abundance of *Proteobacteria* is positively correlated with nonalcoholic fatty liver fibrosis in the normal body mass index population (Jasirwan et al., 2021).

Although the increase in the prevalence of phylum *Proteobacteria* is a sign of an unstable microbial community (dysbiosis) and a possible diagnostic standard of diseases, in a healthy and stable state, the relative abundance of *Proteobacteria* in the intestine sometimes climbs to 45% (average: 2.5% over 15 months) without showing any symptoms of sickness (Shin et al., 2015). Additionally, under certain conditions, some *Enterobacteriaceae* family species and genera, including *Fusobacteriumvarium*, *Staphylococcus*, and *Porphyromonas gingivalis*, are linked to oral pathology, may ectopically colonize the intestine (Kitamoto et al., 2020a). However, when our findings were compared with previous studies, we found a correlation between TMAO and periodontal immunity. Clinical and animal studies have found that periodontitis is often accompanied by increased TMAO levels in peripheral blood (Xiao et al., 2021; Zhou et al., 2022). TMAO has been reported to trigger activated NLRP3 inflammatory corpuscles in a dose-dependent and time-dependent manner to induce vascular inflammation, endothelial dysfunction, and ROS production (Yue et al., 2017; Wu et al., 2020). The NLRP3/caspase-1 signaling pathway is engaged in the IL-1β production in M1 macrophages *in vivo*. NLRP3 inhibitors could significantly reverse this signaling pathway’s activation and improve the symptoms of periodontitis in rats (Sun et al., 2021). In addition, another research showed that TMAO levels were positively correlated with the expression level of IL-1β and hsCRP (Chou et al., 2019). Activated macrophages and lymphocytes produce IL-1β, which is necessary for T cell activation and proliferation, and cooperate with IL-6 to cause periodontal tissue damage and promote periodontitis (Beklen et al., 2007). Similarly, TGF-β not only controls T cell differentiation into regulatory and effector subsets, which is important for the growth and maintenance of immune cells, but it also promotes the production of IL-1, IL-6, as well as TGF-β *via* inflammatory cells, which helps to the onset of periodontitis (Travis and Sheppard, 2014; Leite et al., 2015). Clinical experiments have shown that TGF-β and IL-1β are highly expressed in the gingival tissue of patients with chronic severe periodontitis (Beklen et al., 2007; Chen et al., 2021b). We discovered that in P+TMAO and P groups, IL-1β and TGF-β were significantly higher than in other groups, accompanied by periodontal tissue damage, which is consistent with previous observations. Notably, our results did not rule out the association of TMAO with other factors, for instance, anti-inflammatory cytokines (IL-10) in the pathogenesis of periodontitis. On the contrary, TMAO may work synergistically with various factors to promote the development of periodontitis.

Recent studies have shown the intimate connection between bone metabolism and intestinal microbiota (Sjögren et al., 2012; Yan et al., 2016; Guss et al., 2017). Changes in the intestinal microflora are related to many diseases that lead to bone loss, including malnutrition, IBD, obesity, and metabolic disease (Guss et al., 2017). Animal experiments demonstrated that TMAO activates the ROS-dependent NF-κB signaling pathway to enhance osteoclast polarization and cause bone loss in mice (Li et al., 2019; Wang et al., 2022a). Additionally, TMAO can induce impaired intestinal barrier function, resulting in elevated levels of IL-1β and TGF-β (Nanto-Hara et al., 2020; Li et al., 2021b; Xie et al., 2022; Zhang et al., 2022). Elevated IL-1β and TGF-β circulate to periodontal tissues and damage them. Besides, TGF-β could involve in osteoclastic effects through TLR4/NF-κB signaling (Jiang et al., 2019; Wu et al., 2021). Based on the Micro CT results, our results agree with earlier studies.

**Figure 4:**
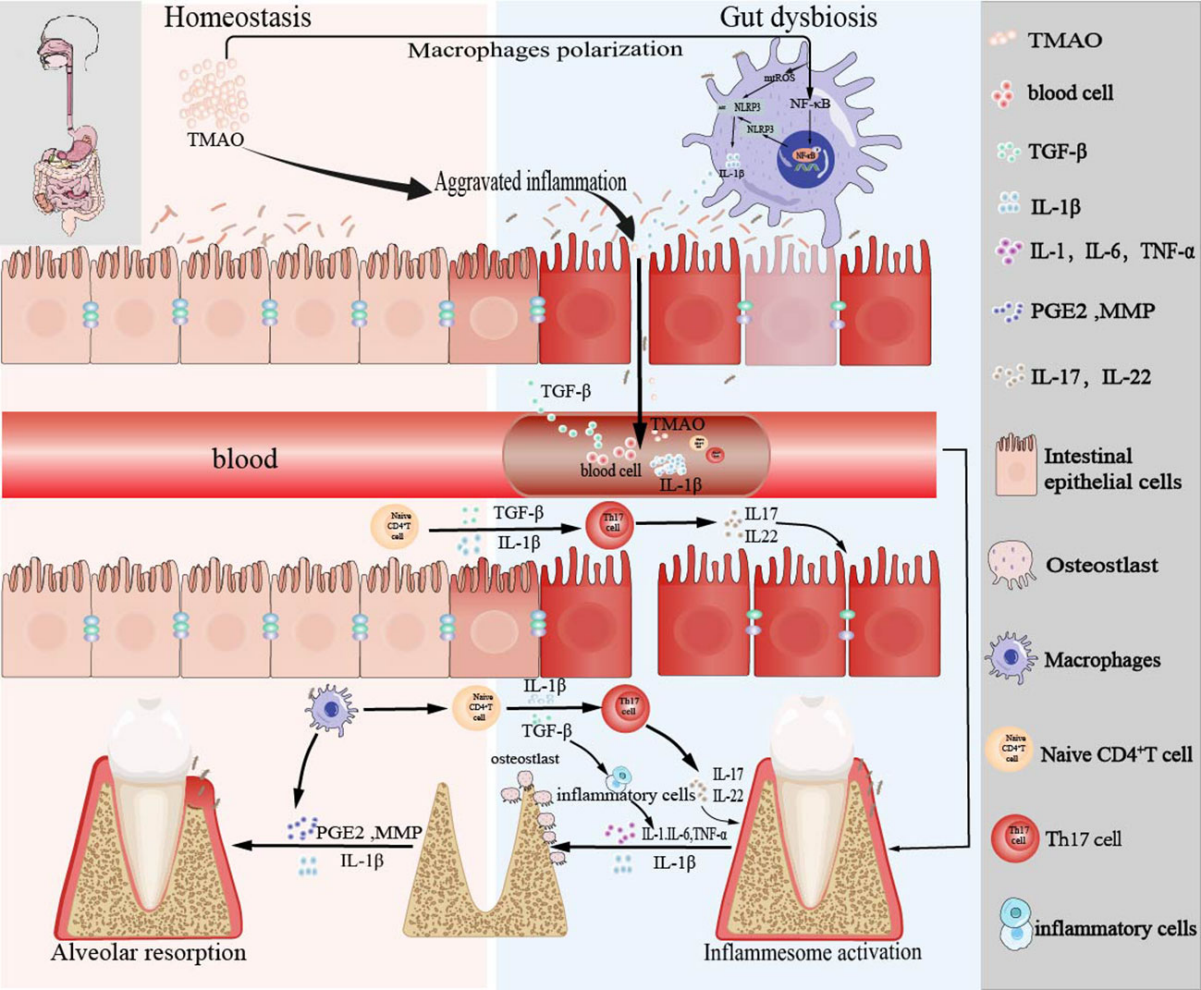
Schema of the bidirectional relationship between periodontal and intestinal immunity affected by TMAO.

In conclusion, as shown in **Figure 4**, our data imply that intestinal inflammation caused by the ecological imbalance of intestinal flora due to exogenous TMAO is related to the progression of periodontitis by involving the regulation of the intestinal-oral axis. However, medical treatment represented by DMB results in the decrease of TMAO level, which can effectively delay the progression of periodontitis and open up a new way for the treatment of periodontitis in the future.

## Data availability statement

The datasets presented in this study can be found in online repositories. The names of the repository/repositories and accession number(s) can be found below: https://www.ncbi.nlm.nih.gov/search/all/?term=PRJNA916071 .

## Ethics statement

The animal study was reviewed and approved by the Institutional Animal Care and Use Committee of Jilin University.

## Author contributions

QW and YS contributed equally to this work. QW and YS conceived and designed the experiments. QW, TZ and CJ conducted the data and bioinformatics analyses. YS, WX and LA conducted the animal experiments. QW, YS, WX and LA wrote the manuscripts. LA and WX oversaw the completion of this study and edited the manuscript. All authors contributed to the article and approved the submitted version.
