# Supplementary material for: Gut microbiota-dependent trimethylamine n-oxide pathway contributes to the bidirectional relationship between intestinal inflammation and periodontitis
Source: Front Cell Infect Microbiol. 2023 Jan 13;12:1125463. doi: 10.3389/fcimb.2022.1125463 (PMC9880481; doi:10.3389/fcimb.2022.1125463)
Supplement: Supplementary file 1 [file DataSheet_1.pdf]

Supplementary Material

Gut Microbiota-Dependent Trimethylamine N-Oxide (TMAO) Pathway Contributes to the Bidirectional Relationship Between Intestinal Inflammation and Periodontitis

Qiqi Wang[1#],Yue Sun[2,3#] , Tianyu Zhou[3] , Cong Jiang[3] , Lan A[2,3*] and Wenzhou Xu[1*]

[1]*Department of Periodontology, School and Hospital of Stomatology, Jilin University, Changchun 130021, China*

[2]*Department of Oral Implantology, School and Hospital of Stomatology, Jilin University, Changchun 130021, China*

[3]*Jilin Provincial Key Laboratory of Sciences and Technology for Stomatology Nanoengineering, Changchun 130021, China*

# These authors contribute equally to this work

*** Correspondence: Lan A:** hialan1983_2001@jlu.edu.cn,

Wenzhou Xu:xuwenzhou@jlu.edu.cn.

Supplementary Figures

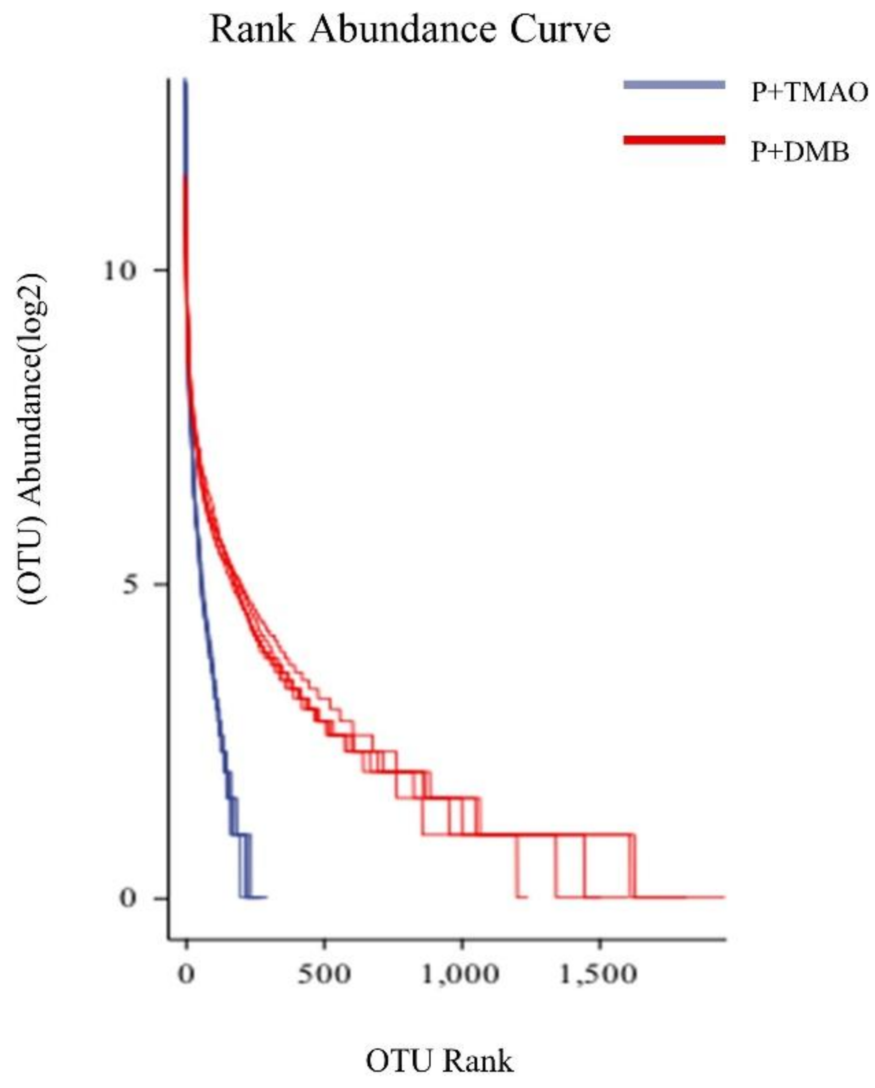

Supplementary Figure 1. The horizontal coordinate is the ordinal number of ASVs/OTUs in order of abundance; the vertical coordinate is the value of abundance/mean value of each ASV/OTU in that sample/group after Log2 log transformation (Log10 transformation, percentage transformation or no transformation); each line represents a sample/group, and the length of the line on the horizontal axis reflects the number of ASVs/OTUs with that abundance in that sample/group number. The gentleness of the fold line reflects the homogeneity of the community composition. The gentler the fold line, the smaller the difference in abundance among ASVs/OTUs in the community and the higher the homogeneity of the community composition, while the steeper the fold line, the lower the homogeneity.

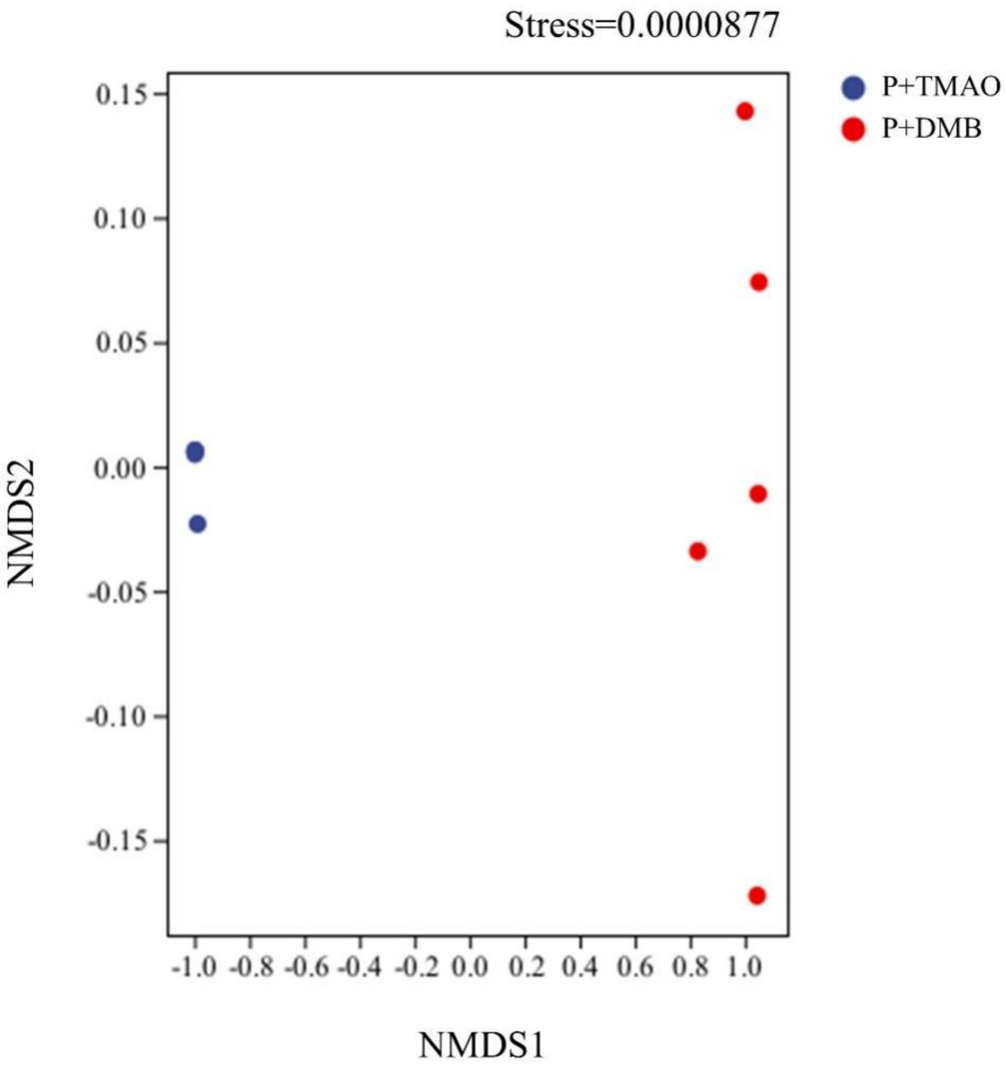

Supplementary Figure2. Each point in the graph represents a sample, and the points of different colors indicate different samples (groups). Since NMDS uses rank ordering, it can be approximated that the closer (farther) the distance between two points, the smaller (larger) the difference between the microbial communities in the two samples.

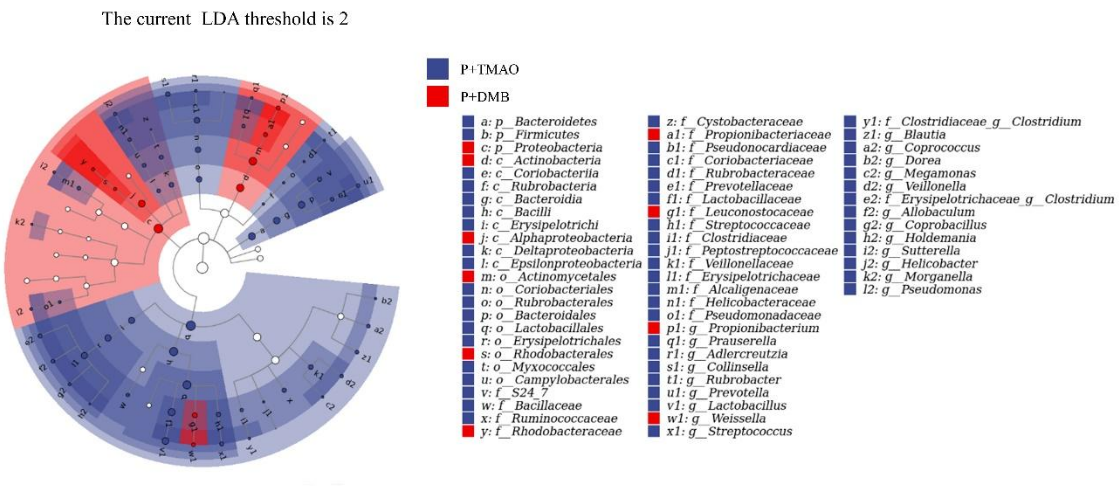

Supplementary Figure3. Taxonomic branching diagrams show the taxonomic rank relationships of the major taxonomic units from phylum to genus (from inner to outer circles) in the sample communities. Node size corresponds to the average relative abundance of that taxonomic unit; hollow nodes represent taxonomic units with insignificant intergroup differences, while nodes in other colors (e.g., green and red) indicate that these taxonomic units exhibit significant intergroup differences and are more abundant in the grouped samples represented by that color. Letters then identify the names of taxonomic units with significant intergroup differences.

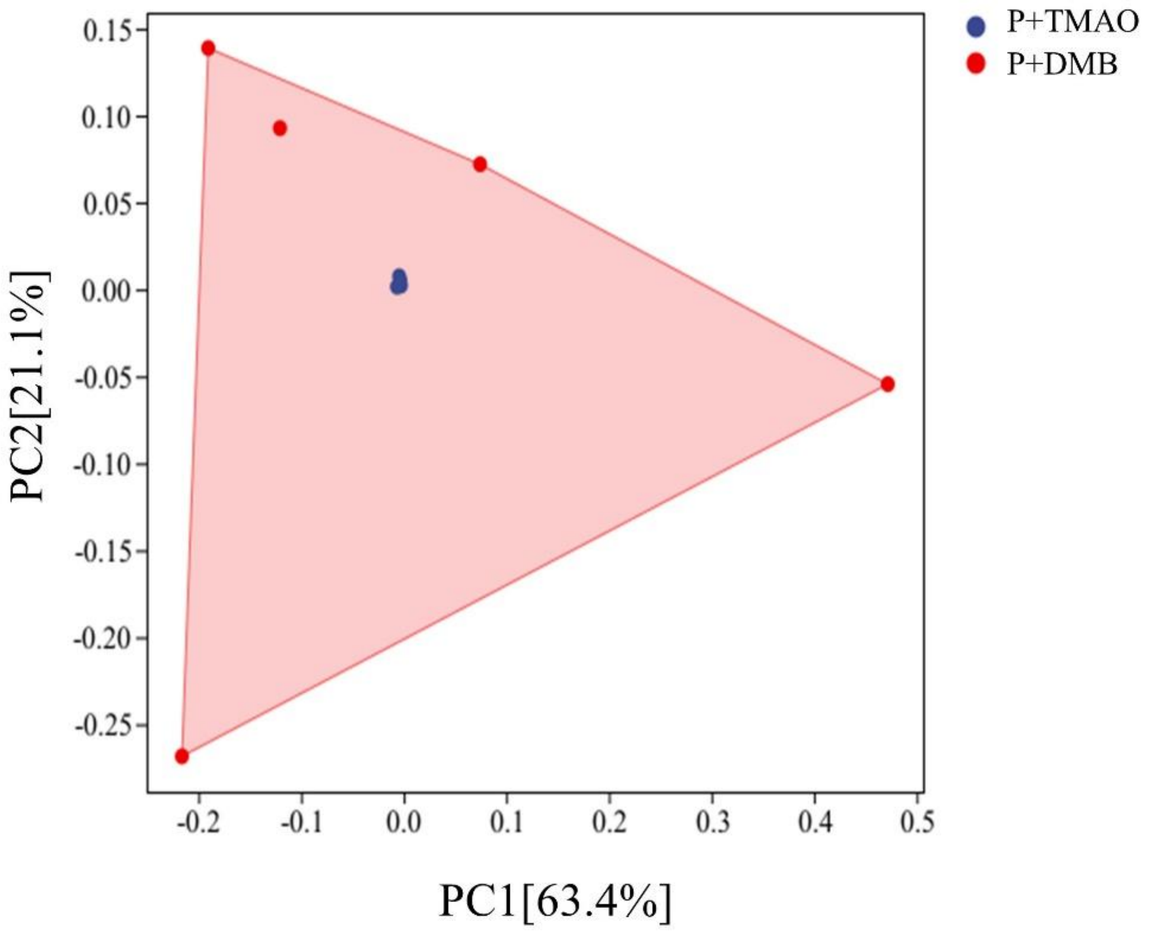

Supplementary Figure3.OPLS-DA plot: each point represents a sample, and points of different colors indicate different groupings. In this case, it is recommended to do a projection analysis, i.e. the closer the projection distance between two points on the axes, the more similar the species abundance composition is between these two samples in the corresponding dimension.
